# Fast and Secure Image Encryption Algorithm with Simultaneous Shuffling and Diffusion Based on a Time-Delayed Combinatorial Hyperchaos Map

**DOI:** 10.3390/e25050753

**Published:** 2023-05-05

**Authors:** Yulin Shen, Jun Huang, Lingling Chen, Tao Wen, Tangyan Li, Guidong Zhang

**Affiliations:** 1 Gansu Computing Center, Lanzhou 730030, China; shenyl@cc.gs.cn (Y.S.); lity@cc.gs.cn (T.L.); 2 College of Information Science and Engineering, Lanzhou University, Lanzhou 730030, China; 120220909111@lzu.edu.cn; 3 Lanzhou Tobacco Company of Gansu Company, Lanzhou 730030, China

**Keywords:** time-delayed hyperchaotic map, fast image-encryption algorithm, simultaneous shuffling and diffusion

## Abstract

Adding time delay to nonlinear systems can significantly improve their performance, making it possible to construct image-encryption algorithms with higher security. In this paper, we propose a time-delayed nonlinear combinatorial hyperchaotic map (TD-NCHM) with a wide hyperchaotic interval. Based on TD-NCHM, we develop a fast and secure image-encryption algorithm that includes a plaintext-sensitive key-generation method and a simultaneous row-column shuffling-diffusion encryption process. Plenty of experiments and simulations demonstrate the superiority of the algorithm in terms of efficiency, security, and practical value in secure communications.

## 1. Introduction

With the rapid development of modern communication and computer technologies, open channel-based communication is widely used in the multimedia-information exchange process. Digital images are a crucial means of conveying multimedia information, and ensuring the security of image data during transmission and storage has garnered significant attention. One effective approach is to encrypt the image at the beginning of transmission and then decrypt it upon acceptance, which can prevent unauthorized access to the original data [1]. However, compared to text files, digital images contain more repeated and closely related data. As a result, algorithms suitable for encrypting text messages, such as DES, AES and RSA, perform poorly when encrypting images. Chaotic systems are characterized by being pseudo-random, sensitive to initial state, unpredictable on trajectory, interval traversal and bounded. These non-linear properties make chaotic systems inherently connected with cryptography. In 1998, J. Fridrich [2] first applied chaotic systems to digital image encryption and proposed the classical image-encryption algorithm with a shuffling-diffusion structure. Since then, chaotic image encryption has received increasing attention from researchers.

Chaotic image-encryption algorithms use two types of chaotic systems: continuous chaos systems and discrete chaos systems. Among these, discrete chaos systems are more suitable for image encryption due to their simplicity, ease of circuit implementation, and low computational complexity [3]. However, some of them are vulnerable to attacks and destruction due to these characteristics. In a study conducted by Arroyo et al. [4], an estimation was made of the trajectories of a one-dimensional chaotic map. The results of their investigation provided compelling evidence that encryption algorithms based on this map are susceptible to security breaches. Similarly, reference [5] reported hidden security problems in the logistic map-based algorithm proposed in [6]. As a result of these security flaws in one-dimensional chaotic systems, researchers have attempted to develop encryption algorithms based on more robust chaotic maps. For example, Zheng et al. [7] proposed an improved cascaded chaotic map 2D-CLSM and a novel encryption scheme based on it and an S-box which is capable of meeting encryption requirements. Furthermore, in reference [8], a universal two-dimensional enhanced cosine coupled model (2D-ECCM) was introduced, which can be used to create new 2D chaotic systems using most of the existing two one-dimensional maps. Using this model, two 2D chaotic systems with better performance were constructed. In addition, Hu et al. [9] designed a 2D-SFCF system by combining two one-dimensional cosine fractional (1-DCFs) systems. It exhibits more complex chaotic behavior and a larger parameter space than 1D chaotic systems, while having a simpler structure than 2D chaotic systems.

Shuffling-diffusion is a typical structure used in chaotic image-encryption algorithms [10,11]. Block image encryption is a process that involves dividing a digital image into sub-blocks with the same number of pixels and then shuffling and diffusing these sub-blocks as a whole. This method is faster than pixel-and-bit encryption algorithms due to its block-level encryption operations, which have less time complexity. Researchers have proposed various techniques to improve the efficiency and security of these algorithms [12]. For example, Zhu et al. [13] developed a fast image encryption that takes the image row (column) as the cyclic encryption unit, and the time overhead is greatly reduced compared with the algorithm taking the pixel as the encryption unit. Xu L [14] presented a new chaotic image-encryption algorithm with a block-image shuffling process and a dynamic index-based diffusion process. This algorithm achieved improvements in efficiency and complexity, but the keystream is not associated with plaintext pixels. Liu W et al. [15] proposed a two-dimensional sinusoidal modulation map (2D-SIMM) with good ergodicity, hyperchaotic behavior, large maximum Lyapunov exponent and high complexity. Based on this map, they combined the shuffling and diffusion processes and proposed a fast image-encryption algorithm that shuffles rows and columns simultaneously. The algorithm is efficient, but the keystream used in the shuffling process is not associated with the plaintext pixels.

The above discussion clearly shows that despite the relatively fast encryption and decryption capabilities of block chaotic image-encryption algorithms, there are still some noticeable problems. First of all, the dynamic properties of the chaotic systems employed in these algorithms are not complex enough. In particular, the discrete or continuous chaotic systems used in these algorithms have narrow hyperchaotic intervals and periodic windows, resulting in shortcomings such as small key space and insufficient randomness. Another drawback is that the keystream used in these encryption algorithms is not inextricably linked to the plaintext, and, thus, cannot effectively thwart selected plaintext attacks. Finally, different mixed-state sequences are used in the shuffling and diffusion phases, which, in turn, leads to a reduction in the usage rate and an increase in the computational complexity and memory footprint of the algorithm.

To address the above problems, this paper designs a time-delayed nonlinear combinatorial hyperchaotic map (TD-NCHM for short) and then presents a fast plaintext-based image-encryption method with simultaneous shuffling and diffusion. The advantages of this method are remarkable. First, TD-NCHM has wider hyperchaotic intervals and complex dynamics without period windows, which can generate random sequences for image-encryption algorithms. Second, the encryption algorithm combines the shuffling and diffusion of rows and columns into a single phase. In particular, the position transformation information of the algorithm is dynamically correlated with the plaintext, which enables the encryption algorithm to effectively resist plaintext attacks. Additionally, this algorithm is capable of fulfilling the encryption requirement with a single round of encryption, thereby significantly reducing its computational complexity. The ability to achieve strong encryption in a single round of computation has important implications for the practical implementation of the algorithm in real-world scenarios, where computational efficiency is a critical factor. By streamlining the encryption process, this algorithm has the potential to offer improved performance compared to conventional encryption methods that require multiple rounds of computation to achieve the same level of security.

The rest of this paper is organized as follows: The TD-NCHM is proposed in Section 2, and its chaotic properties are analyzed and compared with existing chaotic models using bifurcation diagram, trajectory diagrams, Lyapunov exponents, permutation entropy, and the NIST-800-22 test. Section 3 provides a detailed description of the image-encryption algorithm. Additionally, the simulated result of the algorithm is analyzed in Section 4. To conclude this paper, a summary of the results can be found in Section 5.

## 2. A Time-Delayed Nonlinear Combinatorial Hyperchaotic Map

**Figure 1:**
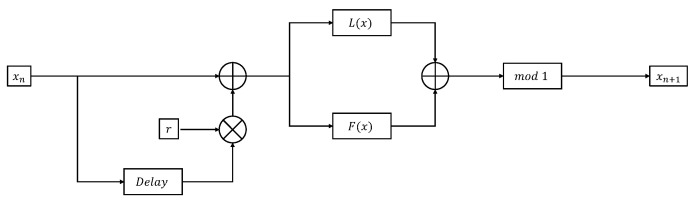
Structure of the TD-NCHM model.

Based on the nonlinear combinatorial map proposed by Zhou Y et al. [16], a new nonlinear combinatorial hyperchaotic model is proposed by adding time delay. The structure of the TD-NCHM model is shown in Figure 1, and the mathematical expression of the model is shown in Equation (1).

(1) $$x_{n+1}=mod\left[F\left(x_{n}+r\times x_{n-1}\right)+L\left(x_{n}+r\times x_{n-1}\right),1\right]$$

The TD-NCHM is constructed based on the one-dimensional chaotic maps F(.) and L(.), where the function mod(x,1) represents the remainder of *x* divided by 1, and the parameter *r* represents the feedback strength of the time delay. By selecting different one-dimensional chaotic maps for F(.), L(.) and altering the feedback strength of the time delay, different chaotic maps can be generated.

In this paper, we chose the one-dimensional logistic map as L(.), the one-dimensional Feigenbaum map as F(.) for constructing the TD-NCHM with a first-order time delay, and its expression is shown in Equation (2):

(2) $$x_{n+1}=mod\left[\mu \left(x_{n}+r\times x_{n-1}\right)\left(1-x_{n}-r\times x_{n-1}\right)+\sin\pi \left(x_{n}+r\times x_{n-1}\right),1\right]$$

where $x_{n}\in \left(0,1\right)$ is output, and μ∈[0,20], r∈[0,20] are the control parameters.

To explore the chaotic performance of TD-NCHM, this paper evaluates it using a bifurcation diagram, trajectory diagram, Lyapunov exponent [17], permutation entropy [18] and NIST-800-22 test [19], and compares it with some recent 2D chaotic systems, such as the Cross 2D hyperchaotic map system (C2HM) [20] and the 2D hyperchaotic map system (2D-HM) [21].

The chaotic system of C2HM is shown in Equation (3).

(3) $$\left\{\begin{array}{c} x_{n+1}=\sin\left[\frac{\alpha }{\sin\left(y_{n}\right)}\right]\\ y_{n+1}=\beta \sin\left[\pi \left(x_{n}+y_{n}\right)\right] \end{array}\right.$$

where with its control parameter α≠0, $\beta \in \left(0,1\right]$, the initial value $y_{0}\neq 0$, the system acquires the best chaotic performance at $a\in \left[0,2\right]$ and $\beta \in \left[0,1\right]$.

The chaotic system of 2D-HM is shown in Equation (4).

(4) $$\left\{\begin{array}{c} x_{n}=\sin\left[\frac{h\pi }{\sin\left(y_{n-1}\right)}\right]\\ y_{n}=r\sin\left(\pi x_{n-1}y_{n-1}\right) \end{array}\right.$$

where *h* and *r* are system parameters and the system acquires the best chaotic performance at $h\in \left[0,7\right]$ and $r\in \left[0,6\right]$.

### 2.1. Bifurcation Diagram Analysis

Bifurcation is a phenomenon in which a dynamic system exhibits a sudden response triggered by a continuous change in one of its parameters, resulting in a change in its motion properties. As the system undergoes continuous bifurcation, it enters a chaotic state from a periodic state. The bifurcation diagram serves to demonstrate the range of parameters in which the system exists in a chaotic state.

**Figure 2:**
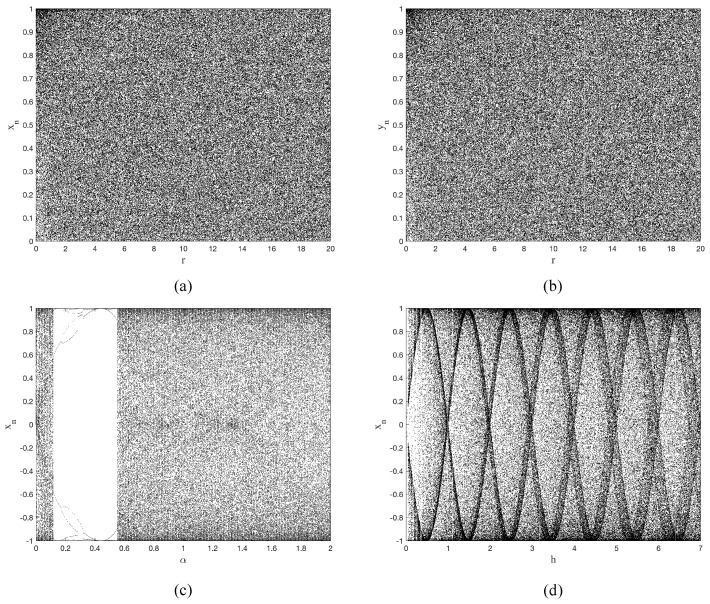
The bifurcation diagrams of chaotic systems. (**a**) The bifurcation diagram of $x_{n}$ in TD-NCHM with μ=4, $r=\left[0,20\right]$. (**b**) The bifurcation diagram of $y_{n}$ in TD-NCHM with μ=4, $r=\left[0,20\right]$. (**c**) The bifurcation diagram of $x_{n}$ in C2HM with $\alpha =\left[0,2\right]$, β=1. (**d**) The bifurcation diagram of $x_{n}$ in 2D-HM with $h=\left[0,7\right]$, r=5.

In this paper, we present the bifurcation diagrams of three chaotic systems as the control parameter increases, as shown in Figure 2. The results indicate that the TD-NCHM demonstrates unique bifurcation features for both output variables across the entire range of parameters. On the other hand, C2HM and 2D-HM exhibit distinct periodic states and uneven distributions in a narrow parameter range. Thus, it is evident that the chaotic range of TD-NCHM possesses the widest coverage, the most extensive and uniform distribution within its parameter range, and the best chaotic performance.

### 2.2. Trajectory Analysis

Trajectory plots are a widely used tool for analyzing the behavior of dynamical systems by visualizing the distribution of their state variables over time. For chaotic systems, the range of the trajectory distribution in a finite domain provides insight into the underlying dynamics of the system. In particular, the width and uniformity of the distribution indicate the dispersion of the chaotic sequence and the strength of the nonlinear effects.

**Figure 3:**
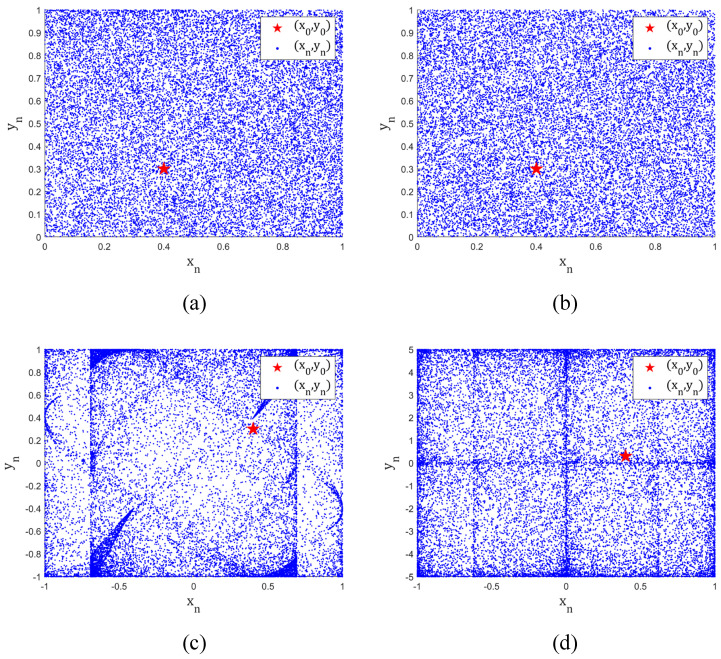
The trajectory diagrams of chaotic systems. (**a**) The trajectory diagram of TD-NCHM with μ=4, r=4. (**b**) The trajectory diagram of TD-NCHM with μ=20, r=20. (**c**) The trajectory diagram of C2HM with α=2, β=1. (**d**) The trajectory diagram of 2D-NM with h=5, r=5.

From Figure 3, it can be observed that the state trajectories of TD-NCHM exhibit a high degree of uniformity within the interval and are spread over the entire range, thus showing a more complex dynamical behavior compared to C2HM and 2D-HM. This characteristic is indicative of TD-NCHM’s ability to generate more dispersed and pseudo-random chaotic sequences. The trajectory plot analysis of TD-NCHM shows that it has highly desirable properties for generating complex and unpredictable chaotic sequences, making it a valuable tool for various applications in the field of chaos theory.

### 2.3. Lyapunov Exponent Analysis

The Lyapunov exponent is a well-established index for quantifying the statistical properties of dynamical systems. A positive Lyapunov exponent indicates the presence of chaos in the system, with the degree of nonlinearity increasing as the maximum Lyapunov exponent increases. In addition, the presence of two positive Lyapunov exponents confirms the existence of hyperchaotic behavior.

**Figure 4:**
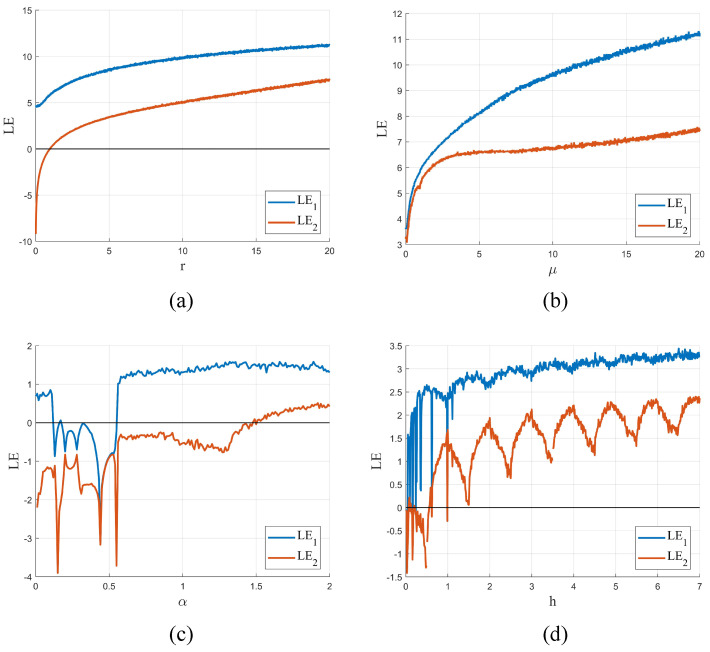
The Lyapunov exponents for chaotic systems. (**a**) The Lyapunov exponent of TD-NCHM with μ=20, $r\in \left[0,20\right]$; (**b**) The Lyapunov exponent of TD-NCHM with $\mu =\left[0,20\right]$, r=20. (**c**) The Lyapunov exponent of C2HM with $\alpha =\left[0,2\right]$, β=1. (**d**) The Lyapunov exponent of 2D-HM with $h=\left[0,7\right]$, r=5.

Based on the results presented in Figure 4, it is clear that all systems exhibit hyperchaotic behavior. However, TD-NCHM shows a wider hyperchaotic interval and a more stable index range within the interval, indicating its potential for use in applications requiring high levels of nonlinear complexity and safety.

### 2.4. Permutation-Entropy Analysis

**Figure 5:**
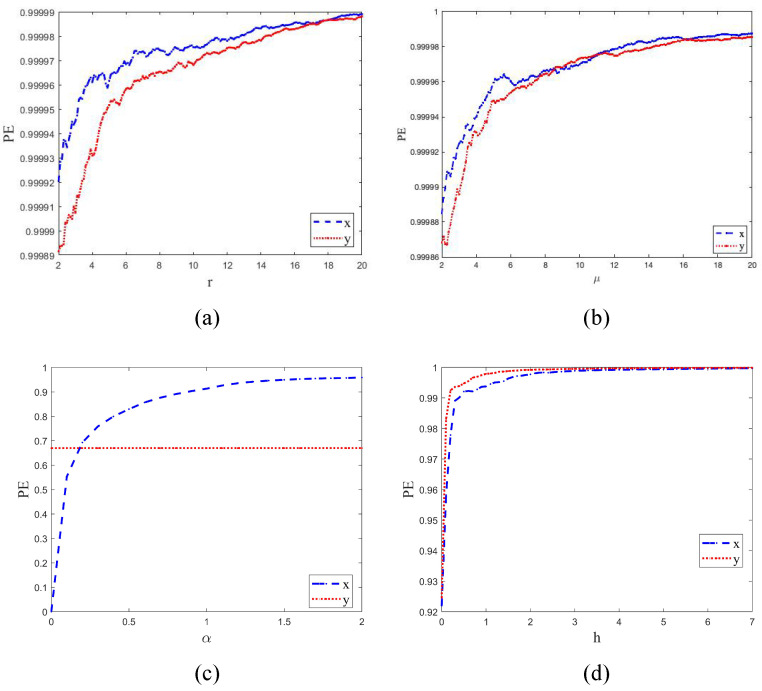
The permutation entropies of chaotic systems. (**a**) The permutation entropy of TD-NCHM with μ=20, $r\in \left[2,20\right]$. (**b**) The permutation entropy of TD-NCHM with $\mu =\left[2,20\right]$, r=20. (**c**) The permutation entropy of C2HM with $\alpha =\left[0,2\right]$, β=1. (**d**) The permutation entropy of 2D-HM with $h=\left[0,7\right]$, r=5.

Permutation entropy is used for measuring the complexity of chaotic sequences. A smaller index corresponds to a more regular and ordered sequence, while a higher index indicates a more complex and disordered sequence. The result of the permutation entropy comparison is shown in Figure 5.

The result shows that TD-NCHM exhibits a permutation entropy that is consistently close to 1 and remains smooth throughout the entire parameter interval, without any significant fluctuations or periodic windows. This observation implies that the TD-NCHM map generates chaotic sequences with more complex nonlinear dynamical behavior and greater randomness over a wider parameter interval. In summary, the comparison of permutation entropies demonstrates that TD-NCHM generates more complex and random chaotic sequences than the other systems considered in this study. This finding highlights the potential of TD-NCHM for applications requiring high levels of randomness and complexity.

### 2.5. Statistical-Randomness Analysis

The NIST-800-22 test suite uses 15 different tests to assess the randomness of generated bit sequences. In this study, 150 binary sequences of length 1,000,000 were generated using the TD-NCHM. The test results were obtained by applying the NIST-800-22 test suite with a significance level of a = 0.01 and are presented in Table 1. For each test, an average *p*-value and a pass rate were calculated. A statistical test is considered passed if the average *p*-value is greater than or equal to 0.01 and the pass rate exceeds 96%. It is clear from the results that the random numbers generated by TD-NCHM pass all the tests with significantly high *p*-values and pass rates approaching 100%.

## 3. A Plaintext Dynamically Related Image-Encryption Scheme Based on TD-NCHM with Simultaneous Shuffling and Diffusion

Classical chaotic image-encryption algorithms typically involve shuffling and diffusion stages, which are iterated multiple times to ensure strong encryption. However, the use of simple shuffling-diffusion operations may compromise the security of the algorithm, while complex or multi-round algorithms slow down the encryption and decryption processes. Moreover, most existing chaotic image-encryption algorithms only employ chaotic sequences to diffuse pixels, bits, or sub-blocks of the plaintext image, without considering the relationship between the plaintext and the chaotic sequence. As a result, these algorithms may not be able to effectively resist plaintext selection attacks.

**Figure 6:**
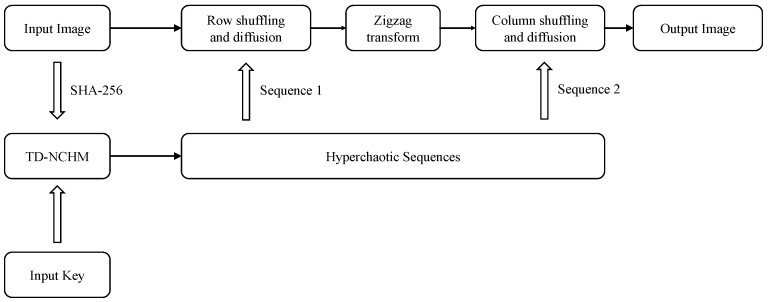
Encryption-algorithm structure.

**Figure 7:**
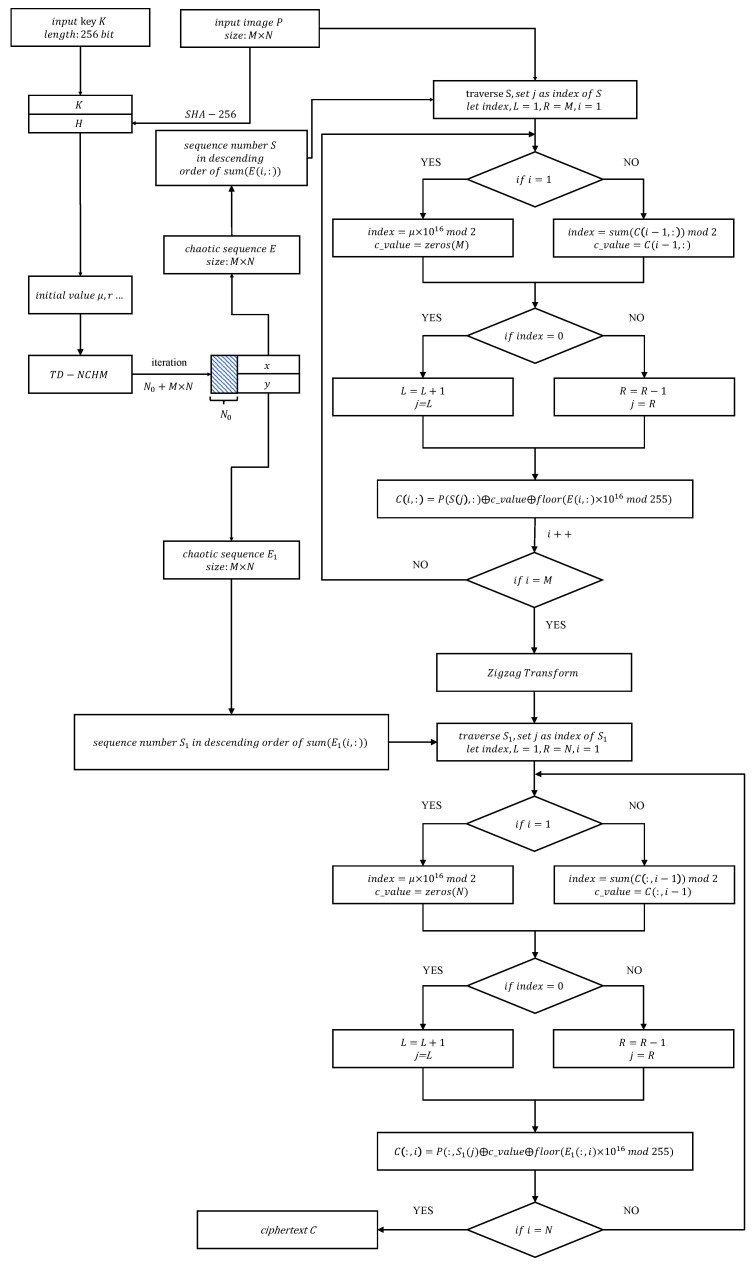
Flowchart of the encryption process.

To address these issues, this section proposes a new plaintext dynamically related chaotic image-encryption scheme based on TD-NCHM with simultaneous shuffling and diffusion. TD-NCHM is a hyperchaotic map that can generate two pseudo-random hyperchaotic sequences in a single run. These sequences are then applied to row shuffling-diffusion and column shuffling-diffusion operations. By diffusing the plaintext in pixel-related shuffling operations, the proposed scheme can effectively resist attacks on the keystream. The structure of the algorithm is presented in Figure 6, and the flowchart of the encryption process is illustrated in Figure 7.

The proposed scheme aims to strike a balance between encryption speed and security, by combining the advantages of simultaneous shuffling and diffusion operations and the use of dynamically related chaotic sequences. The use of TD-NCHM enables the generation of strong chaotic sequences that are dynamically related to the plaintext image, thereby enhancing the resistance of the algorithm to attacks. The simultaneous shuffling and diffusion operations also provide an efficient means of achieving a high level of encryption while minimizing computational complexity. This scheme presents a promising approach for achieving efficient and secure chaotic image encryption.

### 3.1. Initial Value Generation

**Figure 8:**
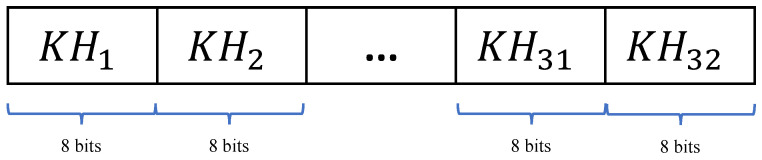
Split the 256-bit KH into $KH_{1},KH_{2},\ldots ,KH_{32}$.

We use a randomly generated 256-bit key as the encryption-algorithm key. In addition, the SHA-256 hash value of the plaintext is used to perturb the key to resist selected plaintext attacks. Based on the key and the hash value, a new initial-value algorithm for chaotic systems is proposed. First, an XOR operation between the 256-bit key *K* and a hash value *H* is performed to obtain a 256-bit binary array KH. Then KH is divided into 32 segments, $KH_{1},KH_{2},\ldots ,KH_{32}$, where each segment consists of 8 bits. The process of splitting KH is shown in Figure 8.

Next, calculate $h_{1},h_{2},h_{3},h_{4}$ by $KH_{1},KH_{2},\ldots ,KH_{32}$, the method is shown in Equation (5), where ⊕ reprints XOR operation.

(5) $$\left\{\begin{array}{c} h_{1}=4\times \frac{KH_{1}⊕\cdots ⊕KH_{8}}{255}\\ h_{2}=h_{1}+3\times \frac{KH_{9}⊕\cdots ⊕KH_{16}}{255}\\ h_{3}=2\times h_{2}+2\times \frac{KH_{17}⊕\cdots ⊕KH_{24}}{255}\\ h_{4}=h_{3}+3\times \frac{KH_{25}⊕\cdots ⊕KH_{32}}{255} \end{array}\right.$$

Finally, μ, *r*, $x_{0}$, $y_{0}$ are calculated with $h_{1}$, $h_{2}$, $h_{3}$, and $h_{4}$. Since the initial values of TD-NCHM have a certain range, an offset is set for the generation to ensure that the initial parameters and initial conditions are within the hyperchaotic interval of the system. In this paper, we set $\bar{\mu }=2$, $\bar{r}=2$, $\bar{x_{0}}=0.2$ and $\bar{y_{0}}=0.3$. The initial values and parameter-generation rules are shown in Equation (6).

(6) $$\left\{\begin{array}{c} \mu =\bar{\mu }+\frac{mod\left[\left(h_{1}+h_{2}\right)\times 10^{16},256\right]}{255}\times 18\\ r=\bar{r}+\frac{mod\left[\left(h_{1}+h_{3}\right)\times 10^{16},256\right]}{255}\times 18\\ x_{0}=\bar{x_{0}}+\frac{mod\left[\left(h_{2}+h4\right)\times 10^{14},256\right]}{255}\\ y_{0}=\bar{y_{0}}+\frac{mod\left[\left(h_{3}+h_{4}\right)\times 10^{14},256\right]}{255} \end{array}\right.$$

### 3.2. Row-Column Shuffling and Diffusion Encryption Method

In order to achieve fast encryption, this paper employs rows and columns as the smallest encryption units and conducts diffusion operations during shuffling. To enhance resistance to chosen plaintext attacks, both the shuffling and diffusion processes are associated with the plaintext pixels, so that any change in a pixel will induce a complete change in the encryption result.

The encryption process consists of two stages. The first stage is the row shuffling-and-diffusion process, where the pixel to be shuffled is determined by the chaotic sequence and the previous diffusion result, and the diffusion result is determined by the chaotic sequence and the row pixel values after shuffling. The second stage is the column shuffling and diffusion process, which follows the same principle as the first stage but utilizes a different chaotic sequence. The encryption scheme is described in detail as follows.

Input: The plaintext image file *F*, a randomly generated key *K*.

Step 1: Let *P* be the pixel matrix size of M×N. Initialize a matrix *C* with the same size as *P* to store encryption results.

Step 2: A hash value *H* is obtained from the image *F* using SHA-256 algorithm, and KH is obtained by bitwise XOR with *K*. Then the initial values $I=\{\mu ,r,x_{1},y_{1}\}$ are generated according to the initial value-generation rules.

Step 3: The *K* of control parameters and initial values are input to the TD-NCHM for $N_{0}+M\times N$ iterations. As the iterations are carried out, two sequences of random numbers *X* and *Y* of length $N_{0}+M\times N$ are obtained from the *x* output and *y* output of TD-NCHM, respectively. Both are of type 0 to 1 fractional. Since chaotic systems can have transient effects (i.e., some of the initial outputs lack randomness), the first *N* outputs are removed ($N_{0}$ = 200 is chosen in this paper), the remaining *X* and *Y* are converted into matrices of size M×N, denoted as *E* and $E_{1}$, respectively.

**Figure 9:**
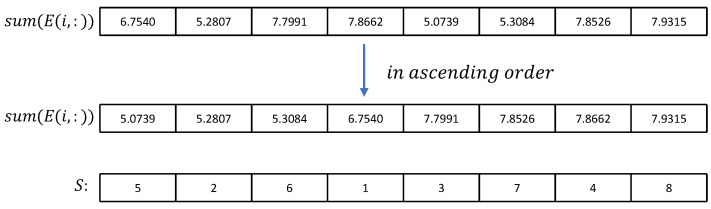
Obtain number sequence *S* from the ascending order sequence of *E*.

Step 4: Sum each row of *E*, then sort the sum result in descending order; the index matrix *S* is obtained. In the same way, $E_{1}$ is summed and sorted by columns to obtain the ordinal matrix $S_{1}$. The length of the matrix *S* is *M* and the length of the matrix $S_{1}$ is *N*. The process of obtaining *S* from *E* is depicted in Figure 9.

Step 5: Row shuffling-and-diffusion encryption. First, a sequence number *i* is selected according to a predetermined rule, where i∈[1,M]. Then, the S(i)-th row of the image *P* is selected as the encryption target. Next, random numbers E(j,:), where j=1,2,…,M, are selected along with the previous encryption result C(j−1,:) (if it exists) to encrypt the selected row P(S(i),:). The encrypted result is stored in the ciphertext matrix C(j,:).

The sequence number i is selected in a manner determined by a 0–1 value called index, if index = 0, the first unused value is selected incrementally from 1, if index = 1, the first unused value is selected decrementally from M. The value of the index is recalculated after each use and is calculated as follows.

(7) $$index=\left\{\begin{array}{c} mod(\mu \times 10^{16},2),\;j=1\\ mod\left[sum\left[C(j-1,:)\right],2\right],\;j>1 \end{array}\right.$$

After selecting the target row to be encrypted according to the rules, encryption is performed using the following. Note that since the elements in *E* are decimals between 0 and 1, the encryption process must be formatted as integers between 0 and 255.

(8) $$C\left(j,:\right)=\left\{\begin{array}{c} P\left[S\left(i\right),:\right]⊕floor[mod\left[E\left(j,:\right)\times 10^{16},256\right]],\;j=1\\ P\left[S\left(i\right),:\right]⊕floor[mod\left[E\left(j,:\right)\times 10^{16},256\right]]⊕C\left(j-1,:\right),\;j>1 \end{array}\right.$$

**Figure 10:**
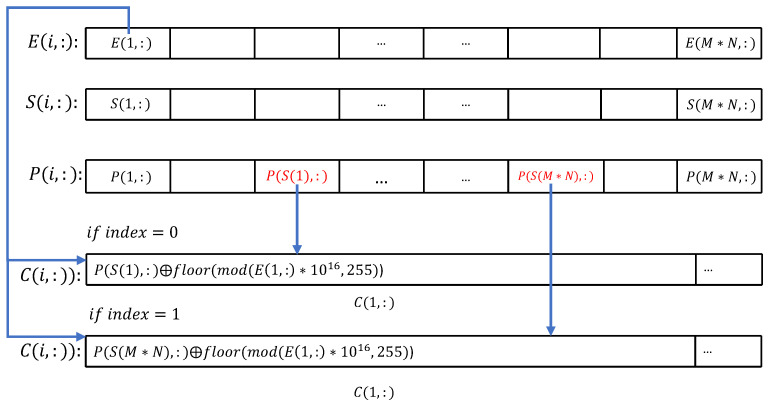
Process of simultaneous shuffling and diffusion.

An example of this process is shown in Figure 10.

**Figure 11:**
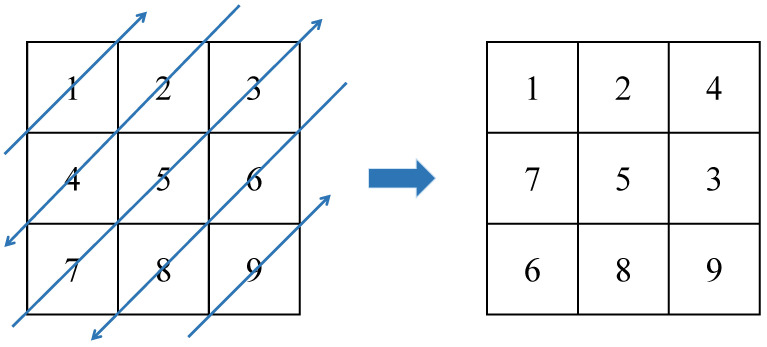
Zigzag transform.

Step 6: Use the zigzag conversion to further enhance the dislocation and diffusion effects. An example of this conversion is shown in Figure 11.

Step 7: Column shuffling-and-diffusion encryption based on *C* obtained. The process differs from Step 5 only in that the row transformation becomes a column transformation, using the sequence $E_{1}$ instead of *E*, and the sequence $S_{1}$ instead of *S*.

As a result, the traversal direction changes dynamically during the traversal process based on the previous encryption result. If the pixel value of a row in the plaintext image changes, the positions of all following rows are changed accordingly.

Algorithm 1 shows the pseudo-code of the row-column shuffling and diffusion encryption method.

### 3.3. Decryption Process

The decryption process is the reversal of the encryption process and is performed as follows.

Input: The cipher images matrix of size M×N, and the shuffled key KH obtained from the plaintext image file.

Step 1: Initialize a two-dimensional matrix *P* size of M×N to store the plaintext. Follow Step 1 in Section 3.2 to obtain the sequence *E*, $E_{1}$, *S*, and $S_{1}$.

Step 2: The method to reverse the diffusion process of the encryption process in iterating through the ciphertext *C*, and use the chaotic sequence $E_{1}$ to perform XOR to decrypt the diffusion process, is shown in Equation (9).

(9) $$C\left(:,j\right)=\left\{\begin{array}{c} C\left(:,j\right)⊕floor[mod\left[E\left(:,j\right)\times 10^{16},256\right]],\;j=1\\ C\left(:,j\right)⊕floor[mod\left[E\left(:,j\right)\times 10^{16},256\right]]⊕C\left(:,j-1\right),\;j>1 \end{array}\right.$$

Step 3: To decrypt the column shuffling process of the encryption process. First, iterate through the sequence $S_{1}$ in the same way as Step 5 in the encryption process. $S_{1}\left(j\right)$, obtained from the iterations, is the position of column *i* in the plaintext. In this way, we can restore the shuffling operation. The restore rule is as follows, where j∈[1,N].

(10) $$P(:,S_{1}\left(i\right))=C\left(:,j\right)$$

Step 4: Inverse the zigzag transformation. The matrix after column decryption is read in the order of inclusion and then column decryption, and then assigned according to the traversal direction of the zigzag transform, and, finally, the image matrix after the inverse transform is obtained.

Step 5: Repeat steps 2 and 3, replacing the operation target with rows instead of columns, and replacing $S_{1}$ with *S*, replacing $E_{1}$ with *E*.

The result of this step is the decrypted plaintext image *P*.
**Algorithm 1** Row-column shuffling and diffusion.**Input:** The plaintext image file *F*.  1:Read the pixel values of image *F* into matrix *P*, with size M×N, initialize a matrix *C* with the same size as *P* to store encryption results;  /* Generate random sequences */  2:H=SHA−256(F), KH=K⊕H, $I=\{\mu ,r,x_{0},y_{0}\}\leftarrow KH$;  3:$N_{0}=200$, X=[],Y=[];  4:**for** i=1 to $N_{0}+M\times N$ **do**  5:    $\left\{x_{i},y_{i}\right\}=TD-NCHM\left(\mu ,r,x_{i-1},y_{i-1}\right)$;  6:    $X\left(i\right)=x_{i}$, $Y\left(i\right)=y_{i}$;  7:**end for**  8:$E=reshape(X\left(N_{0}:end\right),M,N)$, $E_{1}=reshape\left(Y\left(N_{0}:end\right),M,N\right)$;  9:S=sort(sum(E,2)), $S_{1}=sort\left(sum\left(E_{1},1\right)\right)$; /* Row Shuffling and diffusion */  10:left=0, right=M+1, j=1, $C_{l}=\left[\;\right]$;  11:**for** i=1 to *M* **do**  12:       **if** i=1 **then**  13:             $index=mod(u\times 10^{16},2)$;  14:       **else**  15:             $index=mod[sum\left(C_{l},:\right),2]$;  16:       **end if**  17:       **if** index=0 **then**  18:             j=left+1;  19:       **else**  20:             j=right−1;  21:       **end if**  22:       $C\left(i,:\right)=P\left[S\left(k\right),:\right]⊕floor\left[mod\left[E\left(i,:\right)\times 10^{16},255\right]\right]⊕C_{l}$;  23:       $C_{l}=C\left(i,:\right)$;  24:**end for** /* Zigzag transform */  25:C=Zigzag(C); /* Column Shuffling and diffusion */  26:left=0, right=N+1, j=1, $C_{l}=\left[\;\right]$;  27:**for** i=1 to *N* **do**  28:       **if** i=1 **then**  29:             $index=mod(u\times 10^{16},2)$;  30:       **else**  31:             index=mod[sum[C(i−1),:],2];  32:       **end if**  33:       **if** index=0 **then**  34:             j=left+1;  35:       **else**  36:             j=right−1;  37:       **end if**  38:       $C\left(:,i\right)=C\left[:,S_{1}\left(k\right)\right]⊕round\left[mod\left[E_{1}\left(:,i\right)\times 10^{16},255\right]\right]⊕C_{l}$;  39:       $C_{l}=C\left(:,i\right)$;  40:**end for****Output:** The cipher image file *C*.

## 4. Security Analyses and Experimental Results

### 4.1. Simulation Results

**Figure 12:**
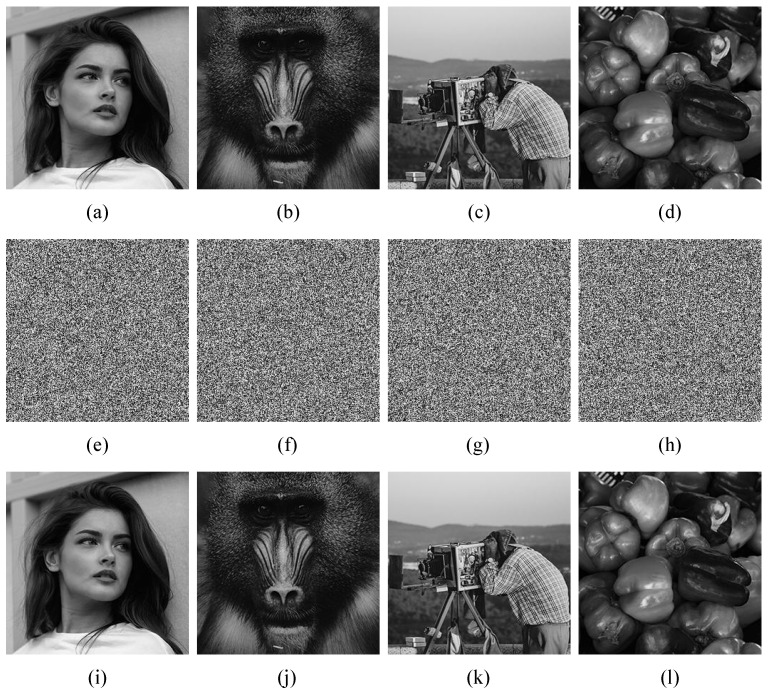
Simulation results. (**a**–**d**) the original images of Girl, Baboon, Cameraman, and Peppers, respectively. (**e**–**h**) the encrypted images of Girl, Baboon, Cameraman, and Peppers, respectively. (**i**–**l**) the decrypted images of Girl, Baboon, Cameraman and Peppers, respectively.

In this study, the test images used for evaluation were Girl, Baboon, Cameraman, and Peppers. The effectiveness of the proposed encryption algorithm was assessed by examining the experimental results, as illustrated in Figure 12. The encrypted images were observed to exhibit a random distribution of pixels, and the decryption process produced clear and complete reconstruction of the original images, indicating the success of the algorithm.

### 4.2. Key-Space and Key-Sensitivity Analyses

A secure and effective encryption algorithm requires the support of a sufficiently large key space. The key of this algorithm consists of 256 binary digits, so its key space is $2^{256}$. Due to current computer hardware conditions, this key has enough space to resist brute-force cracking.

**Figure 13:**
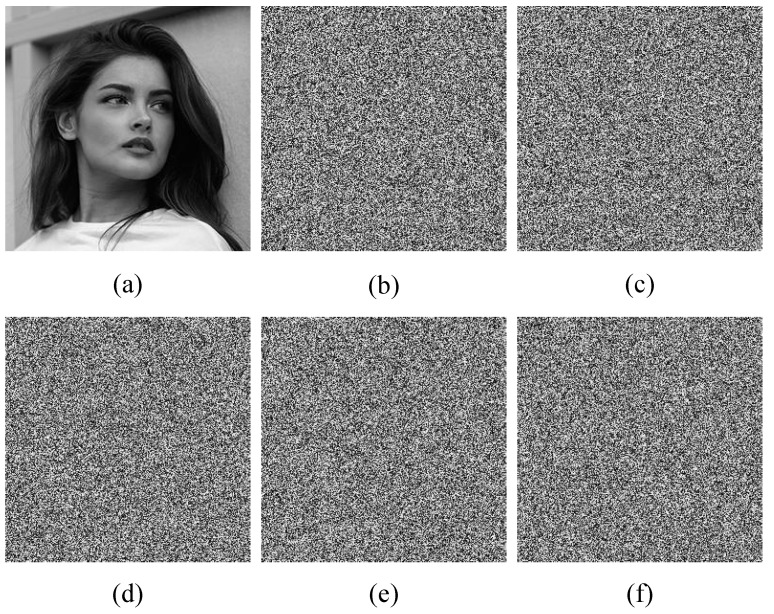
Sensitivity test results of the Girl image. (**a**) The decrypted result with the correct key. (**b**–**f**) The decrypted results with a key that was randomly changed by one bit.

To evaluate the sensitivity of the proposed algorithm to key changes, we randomly altered one bit of the 256-bit secret key and attempted to decrypt the encrypted image using the modified key. The decryption results are presented in Figure 13, which shows that the decrypted images are completely distorted and do not resemble the original ones at all. This confirms that the proposed algorithm is highly sensitive to key changes and can effectively resist attacks on the key.

### 4.3. Histogram Analyses

**Figure 14:**
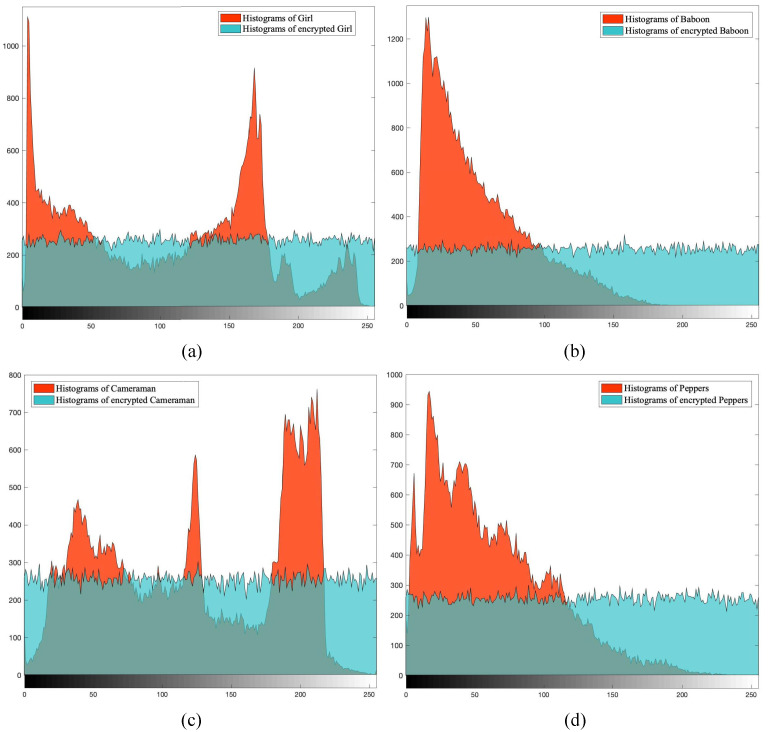
Histograms. (**a**) The histograms of Girl and encrypted Girl. (**b**) The histograms of Baboon and encrypted Baboon. (**c**) The histograms of Cameraman and encrypted Cameraman. (**d**) The histograms of Peppers and encrypted Peppers.

The histograms of the original images and the encrypted images are displayed in Figure 14. The histograms depict the pixel-value distributions of the images. The original images have regular pixel-value distributions, with concentrated features at some pixel values and scattered features at others. In contrast, the encrypted images show a distribution of pixel values that is close to uniform. This indicates that the encryption process disrupts the regularity of the original images and effectively resists statistical attacks on pixel values.

In addition, we use the Chi-square test to quantitatively evaluate the distribution of pixels in the ciphertext. The Chi-square test formula is as follows.

(11) $$\chi^{2}=\sum_{i=0}^{255}\left(\frac{E_{i}-P}{P}\right)$$

where $E_{i}$ and *P* represent the expected and actual frequency values for each gray value, respectively. A smaller value of $\chi^{2}$ indicates a more uniform distribution of gray values in the image. For a 256-level grayscale image, with the confidence interval set at 0.05, the critical value of $\chi^{2}$ is 293.2478. As long as $\chi^{2}$ does not exceed the critical value, it is considered to pass this test.

Table 2 shows the values of $\chi^{2}$ on different images. From the table, it can be seen that for the encryption algorithm proposed in this paper, the $\chi^{2}$ values of all the images are less than the critical value, indicating that the images encrypted using the algorithm in this paper accept the assumption of random-like images, i.e., the pixel distribution is uniform. This shows that the method proposed in this paper has strong resistance to statistical analysis.

### 4.4. Correlation-Coefficient Analyses

The images in their original state typically exhibit high degrees of correlation between adjacent pixels, resulting in the presence of significant meaningful information. However, a key objective of image encryption is to disrupt the correlations between neighboring pixels of the original images. In this regard, this paper employs Equation (12) as the method of calculating the correlations between the pixels of the images.

(12) $$\rho_{xy}=\frac{E\left[\left[x-E(x)][y-E(y)\right]\right]}{\sqrt{D(x)}\sqrt{D(y)}}$$

where $E\left(x\right)=\frac{1}{l}\sum_{i=1}^{l}x_{i}$ and $D\left(x\right)=\frac{1}{l}\sum_{i=1}^{l}{\left[x_{i}-E\left(x\right)\right]}^{2}$ represent the mean and the variance in *l* pixels, respectively.

In this study, 10,000 pixels selected randomly from the horizontal, vertical, and diagonal directions are utilized to compute image correlations. The calculated correlation coefficients before and after image encryption are compared in Table 3. The purpose of image encryption is to break the high degree of correlation between neighboring pixels in the original images. The results in Table 3 indicate that the relationship between neighboring pixels is significantly reduced after encryption, rendering the resulting images with almost no discernible regularity.

**Figure 15:**
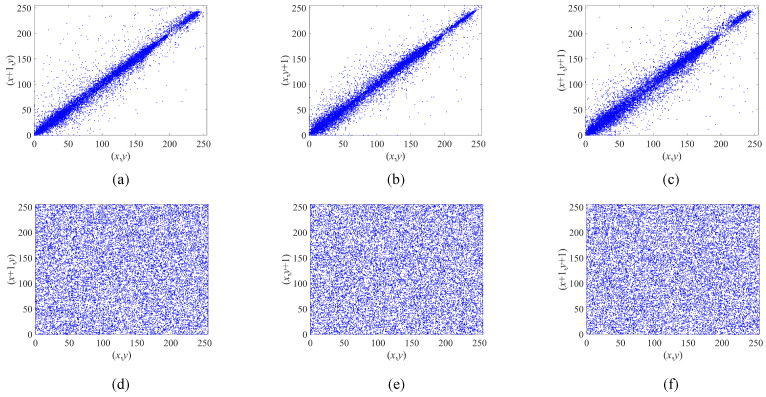
The distributions of adjacent pixels in the original image and encrypted image of Lena. (**a**–**c**) The distributions of the original image in the horizontal, vertical, and diagonal directions, respectively. (**d**–**f**) The distributions of the encrypted image in the horizontal, vertical, and diagonal directions, respectively.

Figure 15 shows the distributions of the neighboring pixels of the original and encrypted images. The results indicate that the pixels in the original images are clustered around the line, implying a strong correlation between neighboring pixels in all three directions. Conversely, the encrypted image exhibits a uniform distribution of pixels across the entire interval. This outcome can be attributed to the disturbance and alteration of the construction and display pattern of the plaintext image during the encryption process. Consequently, statistical attacks are unable to extract sufficient information from the encrypted image due to the disrupted patterns and correlations, thereby ensuring the security and robustness of the proposed encryption algorithm.

### 4.5. Information-Entropy Analyses

In the context of image encryption, the information entropy value of an image is a measure of the degree of randomness or confusion among its pixels. Specifically, a higher entropy value of the encrypted image implies a more effective encryption result. The formula for calculating the information entropy is as follows:

(13) $$H\left(m\right)=\sum_{i=0}^{2^{n}-1}p\left(m_{i}\right)\log_{2}\frac{1}{p(m_{i})}$$

In the case of gray images, the theoretical upper limit of information entropy is 8. Table 4 presents the information-entropy values of the original and encrypted images. The results reveal that the information-entropy value of the encrypted image is in close proximity to the theoretical maximum, indicating a significant degree of pixel confusion in the encrypted image. Furthermore, a comparative analysis reveals that the proposed algorithm yields the smallest difference between the obtained information entropy and the theoretical maximum among all the comparison results. These findings suggest that the proposed algorithm is effective in achieving a high level of encryption by significantly disrupting the pixel patterns and randomness of the original image.

### 4.6. Differential-Attack Analyses

Differential attacks are a class of attacks that target encryption algorithms and keys by analyzing the propagation of changes in plaintext images with slight variations after encryption. To mitigate the risk of differential attacks, encryption algorithms should aim to minimize the similarity between the plaintext and ciphertext images. In this study, three metrics, namely, the Number of Pixel Changes Rate (NPCR), the Unified Average Change Intensity (UACI) [32], and the Block Average Change Intensity (BACI) [33], are employed to quantify the similarity between two images. These metrics are calculated as follows:

(14) $$NPCR\left(P,C\right)=\sum_{i=1}^{M}\sum_{j=1}^{N}\frac{D(i,j)}{M\times N}\times 100$$

(15) $$UACI\left(P,C\right)=\sum_{i=1}^{M}\sum_{j=1}^{N}\frac{\left|P(i,j)-C(i,j)\right|}{M\times N\times 255}\times 100$$

(16) $$BACI\left(P,C\right)=\frac{1}{\left(m-1)(n-1\right)}\sum_{i=1}^{m-1}\sum_{j=1}^{n-1}\frac{m_{ij}\left(P,C\right)}{255}$$

where

(17) $$D\left(i,j\right)=\left\{\begin{array}{c} 0,P(i,j)=C(i,j)\\ 1,P(i,j)\neq C(i,j) \end{array}\right.$$

(18) $$m_{i,j}\left(P,C\right)=\frac{1}{6}\sum_{l=1}^{3}\sum_{k=l+1}^{4}\left|d_{(i,j),l}-d_{(i,j),k}\right|$$

(19) $$d_{(i,j),1}=P_{i,j}-C_{i,j},d_{(i,j),2}=P_{i,j+1}-C_{i,j+1}$$

(20) $$d_{(i,j),3}=P_{i+1,j}-C_{i+1,j},d_{(i,j),4}=P_{i+1,j+1}-C_{i+1,j+1}$$

The theoretical values of these metrics for grayscale images are 99.6094%, 33.4635%, and 26.7712%, respectively. To evaluate the effectiveness of the algorithm against differential attacks, a pixel is randomly selected from the images and its least significant bit is XORed with 1 to modify its value. The modified images are then encrypted to compute the NPCR, UACI, and BACI. This process is repeated 150 times and the results are shown in Table 5.

It can be seen from the table that the mean, maximum, and minimum values of NPCR, UACI, and BACI are very close to the theoretical values, indicating that the proposed algorithm has a high and stable resistance to differential attacks for each image in the dataset. Thus, it is verified that the proposed image-encryption scheme is effective in resisting differential attacks.

Table 6 presents the comparison result of the Lena image with other existing image-encryption algorithms. The comparison is based on the NPCR, UACI, and BACI metrics. As can be observed from the table, the gaps between the NPCR and UACI indices of our algorithm and their theoretical values are smaller than those of other algorithms. Moreover, the difference between the BACI value of our algorithm and its standard value is only 0.0083, indicating that our algorithm outperforms other algorithms in resisting differential attacks.

### 4.7. Encryption-Efficiency Analyses

When evaluating an encryption algorithm, it is important to consider not only its security performance but also its efficiency. In this paper, we use three metrics to measure the efficiency of the proposed algorithm, namely, encryption time, encryption throughput (ET), and the number of machine cycles. The ET and the number of machine cycles are defined as follows:

(21) $$ET=\frac{image_{size}\left(byte\right)}{encryption_{time}\left(second\right)}$$

(22) $$machine\;cycles=\frac{CPU_{speed}\left(Hertz\right)}{ET(byte)}$$

The experimental environment comprises MATLAB R2016a, Inter(R) Core (TM) i7-7700HQ CPU @ 2.80 GHz and 16 GB RAM on Windows 10. Encryption time was computed as the average value after 100 encryptions of the Lena image. Table 7 presents a comparison of encryption time among different algorithms for different image sizes. The results show that for varying input image sizes, the algorithms proposed in this paper exhibit the least time taken. This indicates that the proposed algorithm has high encryption efficiency for the same amount of work.

Table 8 presents the comparisons of encryption throughput (ET) and the number of machine cycles between our proposed algorithm and other algorithms for Lena image of size 256 × 256. It is evident from the table that our algorithm performs well in terms of ET and machine cycles when compared with other algorithms.

## 5. Conclusions

This paper proposes a new nonlinear combinatorial hyperchaotic map named TD-NCHM with time delays and evaluates its dynamics using various methods such as a bifurcation diagram, trajectory diagram, Lyapunov exponent, permutation entropy, and NIST-800-22 test. The results indicate that TD-NCHM has a well-distributed dispersed trajectory, a wider hyperchaotic interval, a larger maximum Lyapunov exponent, and a stable higher permutation entropy exponent. Based on TD-NCHM, a plaintext dynamics-based image-encryption algorithm is presented, which utilizes simultaneous row-column shuffling and diffusion. The proposed algorithm reduces the number of encryption rounds and dynamically associates all shuffling and diffusion with plaintext pixels, thereby enhancing its resistance to attacks. Moreover, the entire encryption simulation requires only one round of operation, thus increasing the encryption efficiency. The experimental results demonstrate that the encryption algorithm has a complex key structure, is sensitive to the keystream, and can withstand brute-force cracking, differential attacks, chosen-plaintext attacks, and chosen-ciphertext attacks. It also excels in encryption speed and efficiency.

## Figures and Tables

**Table 1:** NIST 800-22 test results.

Test	*p*-Value		Pass Rate		Results	
	x	y	x	y	x	y
Frequency	0.726503	0.494392	98.66%	99.33%	Passed	Passed
Block frequency	0.419021	0.902420	98.66%	98.00%	Passed	Passed
Cumulative sums	0.253551	0.589183	99.33%	99.66%	Passed	Passed
Runs	0.991468	0.588652	100%	99.33%	Passed	Passed
Longest run	0.137282	0.935716	98.66%	98.66%	Passed	Passed
Binary matrix rank	0.791880	0.262249	97.33%	100%	Passed	Passed
Discrete Fourier transform	0.534146	0.574903	98.66%	98.66%	Passed	Passed
Non-overlapping template	0.961593	0.902420	99.33%	98.66%	Passed	Passed
Overlapping template	0.883171	0.893001	100%	99.33%	Passed	Passed
Maurer universal statistical	0.699313	0.171867	98.66%	98.66%	Passed	Passed
Approximate entropy	0.319084	0.561227	98.00%	99.33%	Passed	Passed
Random excursions	0.425817	0.953553	98.87%	100%	Passed	Passed
Random excursions variant	0.919445	0.980883	96.62%	100%	Passed	Passed
Serial	0.198690	0.942895	99.33%	98.00%	Passed	Passed
Linear complexity	0.104578	0.935716	100%	100%	Passed	Passed

**Table 2:** Chi-square test results for various images.

Image	$\chi^{2}$ (Plain)	$\chi^{2}$ (Cipher)	*p*-Value (Cipher)	Result (Cipher)
Girl	40,416.5367	238.8003	0.1419	H = 0; Passed
Baboon	106,079.5250	243.1576	0.4218	H = 0; Passed
Cameraman	34,735.4844	228.0975	0.5469	H = 0; Passed
Peppers	62,194.0781	207.9058	0.6324	H = 0; Passed
Lena	40,416.5000	258.6743	0.1847	H = 0; Passed

**Table 3:** Comparison of correlation coefficient values with existing schemes for various images.

Algorithm	Image	Horizontal		Vertical		Diagonal	
		Plain	Cipher	Plain	Cipher	Plain	Cipher
Proposed	Girl	0.9853	0.0022	0.9853	0.0037	0.9853	−0.0013
	Baboon	0.8470	0.0017	0.829	0.0031	0.7757	−0.0018
	Cameraman	0.9227	0.0027	0.9007	−0.0024	0.9058	0.0035
	Peppers	0.9612	0.0010	0.9696	0.0031	0.9436	−0.0015
	Lena	0.9557	0.0011	0.9276	−0.0017	0.8845	0.0007
Ref. [22]	Lena	-	0.0013	-	−0.0049	-	0.0057
Ref. [23]	Lena	-	0.0081	-	0.0065	-	0.0182
Ref. [24]	Lena	-	0.0024	-	0.0009	-	0.0016
Ref. [25]	Lena	-	0.0040	-	−0.0035	-	0.0010
Ref. [26]	Lena	-	0.0083	-	−0.0021	-	−0.0025

**Table 4:** Comparison of information entropy values with existing schemes for various images.

Algorithm	Image	Information Entropy	
		Plain	Cipher
Proposed	Girl	7.6608	7.9979
	Baboon	6.9172	7.9982
	Cameraman	7.5988	7.9977
	Peppers	7.2571	7.9980
	Lena	7.5151	7.9979
Ref. [27]	Lena	-	7.9914
Ref. [28]	Lena	-	7.9973
Ref. [29]	Lena	-	7.9973
Ref. [30]	Lena	-	7.9976
Ref. [31]	Lena	-	7.9974

**Table 5:** The NPCR, UACI, and BACI values of various images.

Image	Type	Min (%)	Max (%)	Mean (%)
Girl	NPCR	99.5529 (−0.0565)	99.6643 (+0.0549)	99.6044 (−0.0050)
	UACI	33.3037 (−0.1598)	33.7188 (+0.2550)	33.4644 (+0.0009)
	BACI	26.6286 (−0.1426)	26.9439 (+0.1727)	26.7698 (−0.0014)
Baboon	NPCR	99.5544 (−0.0550)	99.6704 (+0.0610)	99.6063 (−0.0031)
	UACI	33.3030 (−0.1605)	33.7193 (+0.2558)	33.4579 (−0.0056)
	BACI	26.6267 (−0.1445)	26.9442 (+0.1730)	26.7713 (+0.0001)
Cameraman	NPCR	99.5483 (−0.0611)	99.6689 (+0.0595)	99.6062 (−0.0032)
	UACI	33.3036 (−0.1599)	33.7189 (+0.2554)	33.4696 (+0.0061)
	BACI	26.6296 (−0.1416)	26.9439 (+0.1727)	26.7789 (+0.0077)
Peppers	NPCR	99.5529 (−0.0565)	99.6689 (+0.0595)	99.6093 (−0.0001)
	UACI	33.3034 (−0.1601)	33.7192 (+0.2557)	33.4567 (−0.0068)
	BACI	26.6276 (−0.1436)	26.9435 (+0.1723)	26.7638 (−0.0074)
Lena	NPCR	99.5483 (−0.0611)	99.6765 (+0.0671)	99.6103 (+0.0009)
	UACI	33.3033 (−0.1602)	33.7192 (+0.2557)	33.4658 (+0.0023)
	BACI	26.6288 (−0.1424)	26.9440 (+0.1728)	26.7726 (+0.0014)

The numbers in parentheses indicate the distance from the corresponding theoretical values.

**Table 6:** Comparison of NPCR, UACI, BACI values of various algorithms for Lena image.

Algorithm	NPCR	UACI	BACI
Proposed	99.6103 (+0.0009)	33.4658 (+0.0023)	26.7726 (+0.0014)
Ref. [34]	99.6369 (+0.0275)	33.4335 (−0.0300)	26.8290 (−0.0578)
Ref. [35]	99.6060 (−0.0034)	33.5126 (+0.0491)	26.7603 (+0.0109)
Ref. [36]	99.6000 (−0.0094)	33.5700 (+0.1065)	26.5702 (+0.2010)
Ref. [37]	99.6236 (+0.0142)	33.4898 (+0.0263)	26.7844 (−0.0132)

The numbers in parentheses indicate the gaps from the corresponding standard values.

**Table 7:** Comparison of encryption times required by different encryption algorithms for Lena image.

Image Size	Proposed	Ref. [38]	Ref. [39]	Ref. [40]	Ref. [41]	Ref. [42]
256 × 256	0.0553	0.2695	3.1342	0.4389	0.1690	0.3100
512 × 512	0.2003	1.1869	12.6917	1.8112	0.7080	1.6200
1024 × 1024	0.5547	5.7164	56.0985	7.8457	3.4229	8.2887
2048 × 2048	2.3573	23.4563	229.9568	35.6795	14.1337	40.6077
4096 × 4096	9.6189	107.6835	1066.0248	138.3123	56.5163	220.7666

**Table 8:** Comparison of ETs and machine cycles required by different encryption algorithms for Lena image of size 256 × 256.

Algorithm	ET	Number of Cycles
Proposed	0.9247	2462.04
Ref. [38]	0.2319	10,692.34
Ref. [39]	0.0473	50,405.62
Ref. [40]	0.1650	20,229.45
Ref. [41]	0.2700	10,596.38
Ref. [42]	0.2016	11,904.76

## Data Availability

Data sharing is not applicable to this article as no datasets were generated or analyzed during the current study.
